# Assessment of Cytogenetic Damage and Cholinesterases’ Activity in Workers Occupationally Exposed to Pesticides in Zamora-Jacona, Michoacan, Mexico

**DOI:** 10.3390/ijerph18126269

**Published:** 2021-06-10

**Authors:** Rafael Valencia-Quintana, Rosa María López-Durán, Mirta Milić, Stefano Bonassi, Ma. Antonieta Ochoa-Ocaña, Mayrut Osdely Uriostegui-Acosta, Guillermo Alejandro Pérez-Flores, José Luis Gómez-Olivares, Juana Sánchez-Alarcón

**Affiliations:** 1 Laboratorio “Rafael Villalobos-Pietrini” de Toxicología Genómica y Química Ambiental, Facultad de Agrobiología, Universidad Autónoma de Tlaxcala, CA Genética y Ambiente UATLX-CA 223, Red Temática de Toxicología de Plaguicidas, Tlaxcala 90120, Mexico; prvq2004@yahoo.com.mx (R.V.-Q.); gaperezf@gmail.com (G.A.P.-F.); 2 Laboratorio de Biomembranas, División de Ciencias Biológicas y de la Salud, Universidad Autónoma Metropolitana-Iztapalapa, Ciudad de México 09340, Mexico; gool@xanum.uam.mx; 3 Mutagenesis Unit, Institute for Medical Research and Occupational Health, Ksaverska Cesta 2, 10000 Zagreb, Croatia; mmilic@imi.hr; 4 Department of Human Sciences and Quality of Life Promotion, San Rafaele University, 00166 Rome, Italy; stefano.bonassi@sanraffaele.it; 5 Unit of Clinical and Molecular Epidemiology, IRCCS San Rafaele Pisana, 00166 Rome, Italy; 6 Unidad Académica de Estudios Regionales, Coordinación de Humanidades, UNAM, Jiquilpan 59510, Mexico; antonietao@hotmail.com; 7 Escuela Superior de Ciencias Naturales, Universidad Autónoma de Guerrero Chilpancingo 39105, Mexico; uriosteguiacosta@uagro.mx

**Keywords:** acetylcholinesterase, alkaline comet assay, buccal micronucleus cytome assay, butyrylcholinesterase, cytokinesis-blocked micronucleus assay in lympocytes, DNA damage, personal protective measures

## Abstract

Pesticides have been considered as potential chemical mutagens; however, little is known about toxic and genotoxic effects during pesticide application in Zamora-Jacona, Michoacan State in Mexico. This study sought to determine DNA damage and cholinesterase activities inhibitions in 54 agricultural workers exposed to complex mixtures of pesticides vs. control group (26 individuals) using Comet assay in peripheral whole blood, micronucleus (MN) test in oral mucosa cells, Cytokinesis-blocked MN assay in lymphocytes (L-CBMNcyt) and measuring AChE and BChE activities in whole blood and plasma samples, respectively. Exposed subjects demonstrated significantly elevated levels of primary (Comet assay: tail intensity, tail length, tail moment, Olive tail moment) and permanent DNA damage (MN assay: in blood/buccal cells; frequencies of nuclear buds, binucleated cells, cells with condensed chromatin, karyorrhexis, pyknosis, and karyolysis). However, inhibition of cholinesterase activities (AChE and BChE) was not observed in the workers. Confounding factors including sex, age, BMI, working exposure period, protection level, smoking habit (cigarettes per day units), alcohol consumption (weekly), medication, were considered in the analysis. These combined techniques demonstrated usefulness in the health hazards risks pesticide exposure assessment and suggested the need for periodic monitoring together with the education and the training of occupational workers for the safe application of potentially harmful pesticides.

## 1. Introduction

Zamora-Jacona is an urban area resulting from the conurbation of Zamora and Jacona municipalities, being the third most populous urban nucleus in the State of Michoacán, Mexico. The cultivation of red fruits (strawberry, blackberry, blueberry, and raspberry) is one of the main economic activities and the source of permanent and temporary jobs throughout the agri-food production chain, favoring producers, marketers, industrializers, and consumers. This area is marked as the most important red fruits producer at the national level (covers more than 70% of national production), with a large amount of production dedicated to the export [1,2]. To maintain high levels of production and quality, a growing variety of the chemicals (pesticides) is used in the plant treatment, mostly against mites and botrytis. These species affect the quantity, quality, and growth of the red fruit crops in this urban area, with a characteristic variety in the number of their species [3,4]. Extensive literature reported deleterious effects of pesticides exposure on the environmental and human health, often leading to genetic alterations (genotoxicity, mutagenicity, carcinogenicity, or teratogenicity), neurotoxicity, endocrine disruption, reproductive development, and immunological effects [5,6,7,8]. Reviews of Bolognesi [9], Bull et al. [10], and Bolognesi et al. [11] concluded that exposure to pesticides represents a genotoxic risk, but due to the studies’ limitations and uncertainties, better characterization of exposure, confounding factors, and the appropriate assessment of assays are recommended [12]. Nowadays, due to the simultaneous fight against different pest species, agricultural workers are exposed to pesticide mixtures with different mechanisms of action and, although with the lower pesticide dose currently used than in the past, the effect of long-term exposure on various aspects of human health is not clearly defined [7]. Various biomarkers are used in the biomonitoring of human populations exposed to environmental pollutants, among them the comet (primary DNA damage detection), and the micronucleus (MN) assay (permanent DNA damage detection) have found their place due to their sensitivity in assessing different types of DNA damage. In the occupational setting risk assessment, the evaluation of the acute and chronic exposure done by analyzing cholinesterase activity can complement cytogenetic analysis [13,14]. There are studies reporting genotoxic results or different cholinesterase activity in agricultural workers, but only few combined these methods together [15,16,17,18,19,20], and for sure none of them on Mexican agricultural workers.

Thus, the main objective of this study was to determine whether pesticide exposure of agricultural workers in Zamora-Jacona in Michoacan, Mexico, induced alterations in cholinesterases activity (acetylcholinesterase (AChE) and butyrylcholinesterase (BChE) in whole blood/serum) and in cytogenetic damage using alkaline comet assay. Exposure assessment was then associated to the results of the MN assay in binucleated lymphocytes (circulating through the organism and collecting all type of DNA damage from the exposure, including skin exposure), and of MN assay in human buccal epithelial cells (as the first site of contact with the organism after inhalation or ingestion). The simultaneous use of these techniques enabled us to distinguish and evaluate primary DNA damage induced by recent exposure to xenobiotics and cumulative genome damage as the result of chronic long-term exposure to pesticide and to compare one invasive and one noninvasive technique in permanent DNA damage evaluation.

## 2. Materials and Methods

### 2.1. Study Population and Sample Collection

The State Research Ethics Committee of the Tlaxcala State Bioethics Commission approved the study with the registration number CI-CEI-01/2018. At the beginning of the study, before sample collection, all participants were informed about the objectives and the experimental details of the study, signed an informed consent form and answered a structured questionnaire over socio-demographic characteristics including age, gender, body mass index (BMI, kg/m^2^), alcohol consumption (weekly) and smoking habit (units by day) for confounding factors discrimination, in addition to details about pesticides exposure and exposure period (working period in years), and the use of personal protective measures (PPM). Medication was considered when volunteers reported habitual ingestion of pills or medicaments.

This study was performed on 80 individuals, between 18 and 72 years, from Zamora-Jacona municipalities, in the state of Michoacan, Mexico. Participants were divided into two groups, i.e., subjects occupationally exposed to pesticide mixtures, composed by 54 agricultural workers, and a control group of 26 individuals, selected from the general population of the same geographic area, with no labor activity related to agriculture, nor exposure to any environmental contaminant or genotoxic agent. The number of volunteers varies between two groups, due to the difficulties in the control group sampling, since people were mostly scared from the invasive blood sampling.

Two samples of peripheral blood were collected from each farmer or control subject by trained professionals into vacutainers tube with EDTA anticoagulant (4 mL) for cytogenetic assays and vacutainer tube without anticoagulant (4 mL) for serum cholinesterase activities, respectively. The samples were aliquoted for comet assay, micronucleus assay and cholinesterase assay (in whole blood and in serum).

For buccal samples, the subjects were asked to rinse their mouth with tap water and a sponge head swab was used to obtain the sample of epithelial cells from the buccal mucosa, by rubbing the inside of each one of the participants’ cheeks, with samples collected in a 50 mL tube with cold phosphate saline buffer (PBS, Sigma, St. Louis, MI, USA).

All samples, coded, to avoid possible bias, were transported the same day to the laboratories within 2 h, stored at 4 °C, protected from the light, and were processed as quickly as possible within the same day.

If not reported otherwise, all chemicals were from Sigma Aldrich, St. Louis, MI, USA.

### 2.2. Cholinesterases Activity

The activities of acetylcholinesterase (AChE) and butyrylcholinesterase (BChE) differ in their preferred substrate and sensitivity to inhibitors [21] (Massoulié et al., 1993). Cholinesterases activity in whole blood and serum were spectrophotometrically assayed by Ellman method (1961) [22].

#### 2.2.1. Serum Cholinesterase Activities

Whole blood was collected using vacutainer tubes without anticoagulant (Becton Dickinson, Franklin Lakes, NJ, USA), serum was separated from the clot by centrifugation (1881× *g*, 10 min) and stored at −20 °C until analysis. For AChE activity, serum was assayed with 1 mM acetylthiocholine and 50 µM tetraisopropyl pyrophosphoramide (Iso-OMPA), a specific BChE inhibitor [21]. Serum BChE activity was measured with 1 mM butyrylthiocholine (BuTCh) and 10 µM 1,5-bis(4-allyldimethyl-ammoniumphenyl)-pentan-3-one dibromide (BW284c51), a specific AChE inhibitor [21]. The activities of AChE and BChE were expressed as unit of cholinesterase activity (U), one unit corresponding to the amount of enzyme, which hydrolyzes one µmol of the substrate per hour at 20 °C. To guarantee the accuracy of the value obtained, analyses were done in quadruplicate.

#### 2.2.2. Whole Blood Acetylcholinesterase Activity

From the whole blood sample collected in vacutainer tube with EDTA (Becton Dickinson, Franklin Lakes, NJ, USA) as an anticoagulant. The mixture was incubated at 37 °C for 20 min. After this time, 0.025 mL of acetylthiocholine iodide (28.3 mM) was added; the change in absorbance at 405 nm wavelength was followed for several minutes in spectrophotometer (UNICO, China). One unit (U) of cholinesterase activity is the amount of enzyme, which hydrolyzes one µmol of the substrate per min at 37 °C. Measurements were made in triplicate.

### 2.3. Cytogenetic Assays

#### 2.3.1. Comet Assay

The alkaline comet assay was carried out in the whole blood sample with anticoagulant (EDTA) according to the procedure described previously [23] with some modifications, respecting the recommended MIRCA rules (Minimum Information for Reporting Comet Assay) [24]. Slides were prepared in duplicates per each subject. For slide preparation, 20 µL of blood was mixed with 200 µL of 1.0% (*w/v*) low melting agarose. A100 µL of this mixture was carefully stirred, dropped on an already precoated slide (1% normal melting agarose), and kept on ice during each gel layer polymerization. After delicate coverslip removal, slides were placed in a freshly made cold lysis solution (2.5 M NaCl, 100 mM EDTA, 10 mM Tris, pH 10, 10% DMSO, and 1% Triton X-100 pH 10) for 1 h at 4 °C. After the lysis, DNA on slides was denaturated for 25 min in a freshly made cold electrophoresis buffer solution (300 mM NaOH and 1 mM Na_2_EDTA, pH ≥ 13). Then, horizontal electrophoresis was performed in a new cold electrophoresis buffer solution at 25 V (1 V/cm) and 300 mA for 20 min. Subsequently, slides were rinsed three times in a freshly prepared neutralization buffer (0.4 M Tris, pH 7.5), fixed in the absolute ethanol (Merck Millipore, Bedford, MA, USA) for 5 min, dried at room temperature and stored in a dark and dry container until the analysis. Before the analysis, slides were both rehydrated and stained with 40 μL of ethidium bromide solution (20 μg/mL) for 10 min. For each subject, 100 cells (50 cells per each of two replicate slide) were randomly analyzed on an epifluorescence Axiostar Plus Carl Zeiss microscope connected with Comet Assay IV image analysis system (Instem, London, UK). The parameters scored to determine DNA damage were comet tail length (measured in micrometers), tail intensity (% of DNA in comet tail), tail moment and Olive tail moment (moments are calculated by the software). All the slides were coded before scoring to avoid bias and were scored by one person to avoid interscorer variability.

#### 2.3.2. Cytokinesis-Block MN Assay (L-CBMNcyt)

For cytokinesis-blocked MN assay, cultivation and slide preparation was done according to standard protocol [25]. Whole blood from the vacutainer with anticoagulant (EDTA) (0.5 mL) was added to the medium mixture of 4.5 mL of RMPI 1640 medium, 0.2 mL phytohemagglutinin A (Microlab, Mexico) and 0.1 mL of antibacterial and antifungal solution (Microlab, Mexico) and incubated for 72 h at 37 °C. Cytochalasin B was added to the cultures at a final concentration of 6 μg/mL after 44 h of incubation, to arrest cytokinesis. At 72 h, samples were centrifuged (1254× *g*, 10 min), and 5 mL of cold 0.075 M KCl was added dropwise while vortexing only the pellet. Samples were left for 10 min at room temperature, and after centrifugation, cells in the pellet were fixed in ice-cold methanol: acetic acid (3:1) solution, centrifugation and fixation were repeated three times to clean the pellet and finally from the cell pellet with 1 mL of fixative, few drops of cell suspension were put gently on the slide and air-dried. Slides were made in duplicate from each culture (also in duplicate), were stained with 5% Giemsa (Merck Millipore, Bedford, MA, USA) for 10 min and analyzed using Axiostar microscope (Zeiss, Oberkochen, Germany). For each individual, MN frequency was assessed in 1000 binucleated (BN) cells per replicate (2000 BN in total) and expressed as the frequency on 1000 BN cells. To minimize the variability, the same researcher carried out all the analysis.

#### 2.3.3. Buccal Micronucleus Cytome Assay

Buccal cell samples in PBS were centrifuged at 627× *g* for 10 min at room temperature and pellet drops were applied on clean microscope slides. Smears were air-dried, fixed in methanol/acetic acid (3:1) and stained using the Feulgen reaction technique. The technique was modified as follows: smears were pretreated with 1 N HCl for 10 min at room temperature, placed for 10 min in 1 N HCl at 60 °C, rinsed in distilled water, put in Schiff’s reagent for 90 min, and washed with running tap water. The criteria followed for buccal MN parameters were according to Thomas et al. [26]. Three thousands of epithelial cells were screened for each individual to determine the MN frequency and frequency of other nuclear anomalies such as nuclear buds (NB) (also biomarker of DNA damage), binucleated (BN) cells (biomarker of changed cell proliferation and possible aneuploidy and genomic instability), cells with condensed chromatin (CC) and karyorrhectic (KR) cells (biomarkers of early and late apoptosis-programmed cell death), pyknotic (PK) and karyolitic (KL) cells (biomarkers of early and late necrosis). All these parameters were classified according to Bolognesi and Fenech [25] and Holland et al. [27]. Results were later expressed as the frequency per 1000 BN cells. Slides were also coded before scoring to avoid bias. The analysis was performed using microscope Axiostar Zeiss (Oberkochen, Germany), by an experienced scorer.

### 2.4. Statistical Analysis

Descriptive statistics (mean, standard deviation, standard error), Chi square with Yate’s correction and Mann–Whitney tests were used to compare the data of sociodemographic characteristics. Differences in tail length, tail intensity, tail moment, Olive tail moment; micronucleus and micronucleus abnormalities, in both assays, as well as blood/serum AChE/BChE activity differences, and differences in confounding factors were determined using Mann–Whitney test, this was performed in GraphPad Prism version 6.0 software.

Multiple linear regression analysis was used to evaluate the effect of independent variables (sex, age, BMI, working period in years, protection level, smoking habit (cigarette units by day), alcohol consumption (events by week), medication, on assay parameters as dependent variables (comet assay, micronucleus, binucleated, pyknotic, karyolitic, nuclear buds, condensed chromatin) each model was selected by the stepwise method, to demonstrate the best predictors (independent variables) for dependent variables, analysis was performed with Minitab-19, significance level was *p* < 0.05.

## 3. Results

### 3.1. Sociodemographic Characteristics of the Study Groups

Table 1 shows demographic characteristics of the populations studied (exposed and non-exposed group). The exposed group included 54 agricultural workers, aged between 18 and 71 years (mean age ± standard deviation (SD), 36.09 ± 1.60). The non-exposed group consisted of 26 subjects, between 19 and 72 years of age (37.62 ± 3.10), with no known exposure to genotoxic agents.

The distribution of age (U = 582.2, *p* = 0.642) and BMI by gender (female U = 13, P0.185; male U = 125.0, 0.177) was very similar in both groups. Most agricultural workers in Jacona-Zamora, Michoacan in Mexico were males (96%). We could not match the groups according to the gender, smoking habit nor alcohol consumption, due to the reasons already stated, moreover, the proportional frequency to smoking habit (smoking or no smoking) were not different between exposed and non-exposed group (χ^2^ = 1.575, g.l. = 1, *p* = 0.209). For alcohol consumption, exposed group was different in the distribution of frequency since more than half of the individuals reported alcohol intake, and in non-exposed there was only one reporting participant (χ^2^ = 15.17, g.l. = 1, *p* < 0.0001).

According to the face-to-face questionnaire applied, most pesticide sprayers (87%) described the use of protection (facemask, gloves, overalls, etc.); to avoid direct exposure to complex mixture of used pesticides through inhalation route, or in a contact with skin or eyes.

Table 2 presents the list of the pesticides mostly used among the examined workers. These pesticides are classified from slightly to highly hazardous according to the World Health Organization (WHO) [28], Environmental Protection Agency (USEPA) [29], and International Agency for Research on Cancer (IARC) [30]. Five of the pesticides used by workers in our study have been classified by IARC like probably carcinogenic to humans (Group 2A) (diazinon, malathion, and aldrin metabolite), parathion was classified like possibly carcinogenic to humans (Group 2B), and dicofol was not classifiable as to its carcinogenicity to humans (Group 3). However, WHO classifies these pesticides as extremely hazardous (parathion), highly dangerous (gusathion, tamaron, furadan, lannate, vydate, and baytroid), moderately hazardous (diazinon, dimethoate, lorsban, orthene, dicofol, endosulfan, karate, talstar, and paraquat) and slightly hazardous (malathion). Workers were usually exposed to a complex mixture of pesticides, represented mostly by insecticides, herbicides, and fungicides, principally organophosphates (47%), carbamates (21%), organochlorines (16%) and pyrethoids (16%).

### 3.2. Cholinesterases Activity

The AChE erythrocytes (blood) average activity was for 14% higher from the control and significant from the non-exposed group (4.59 ± 1.23 U/mL, range from 2.04 U/mL to 7.11 U/mL vs. 3.83 ± 1.34 U/mL, range from 1.4 U/mL to 5.87 U/mL, *p* < 0.01) (Table 3).

The AChE serum average activity was also significantly higher when compared to the non-exposed group (49.55 ± 14.68 U/mL, range from 15.55 U/mL to 79.84 U/mL vs. 37.62 ± 12.22 U/mL, range from 20.18 U/mL to 69.07 U/mL, *p* < 0.01) (Table 3).

Similarly, BChE serum average activity was significantly higher in the exposed group (289.80 ± 73.57 vs. 217.93 ± 76.37 U/mL, *p* < 0.003), in a range from 97.78 U/mL to 428.60 U/mL vs. the control range from 97.78 U/mL to 380.40 U/mL (Table 3).

### 3.3. Cytogenetic Damage

#### 3.3.1. Comet Assay

The primary DNA damage results are shown in the Table 4. All the examined parameters, tail length, tail intensity, tail moment and Olive tail moment were significantly increased (*p* < 0.0001) in the exposed group (78.80 ± 3.77 µm, 22.40 ± 1.48%, 6.34 ± 0.76 and 16.29 ± 1.49) when compared to the control group (55.62 ± 2.72 µm, 9.21 ± 0.80%, 1.89 ± 0.24 and 5.69 ± 0.64), clearly demonstrating higher levels of primary DNA damage in the pesticide-exposed subjects, and confirming also recent exposure to the pesticides. We should mention that control group had tail intensity values in the range of the values for a healthy control (up to 11% of tail intensity or arbitrary units of DNA damage is allowed in healthy control, our value was 9.21 ± 0.80%, mean ± standard deviation) [31]. When pesticide workers were divided into two groups based on gender, age (<35 and ≥ 35 years), smoking, and drinking habit, PPE, and duration of work (<6 and ≥ 6 years), no significant differences in the comet parameters between different groups of variable tested were found (*p* > 0.05). However, interestingly, when DNA damage was compared between workers with normal BMI against workers with different degrees of obesity, the latter presented a statistically significant higher tail moment and tail intensity (*p* = 0.006), data not shown. This condition was confirmed with a linear regression model adjusted, and the only one independent variable that explains variation in tail moment was BMI (b = 0.3161 ± 0.156, *p* = 0.049).

#### 3.3.2. Cytokinesis-Block MN Assay (L-CBMNcyt)

In L-CBMNcyt assay in peripheral blood lymphocytes, it was also observed that exposure to pesticides significantly increased the MN frequency as compared to the controls. A mean number of MN per 1000 BN cells was 7.53 ± 0.81 in the exposed workers and 2.68 ± 0.95 in the control group (*p* < 0.001) (Table 4), with 2.88 times higher values in the exposed group. No significant difference in the micronucleus frequencies was found when exposed group was divided in two groups by gender, age (< 35 and >35), BMI, smoking, and drinking habit, duration of work (<6 and ≥6 years), and PPE. In these cases, none of the variables had statistically significant influence on the values of micronucleus frequency.

#### 3.3.3. Buccal MN Cytome Assays

The buccal mucosa cytome assay was the other genotoxicity assay used to evaluate the risk from the exposure to pesticides in agricultural workers and the results are presented in Table 4. In addition to MN, scoring of cells for other nuclear anomalies such as binucleated and condensed chromatin cells, karyorrhexis, pyknosis and karyolysis was also performed. The MN frequencies expressed per 1000 oral mucosa cells were 0.78 ± 0.20 and 0.269 ± 0.072, in the exposed and control groups, respectively, with significant statistical difference (*p* = 0.021). Interestingly, just as in the L-CBMNcyt assay, exposed group had again 2.88 times higher micronucleus values than in the control group. In the same way, the differences in the mean frequency in nuclear abnormalities were statistically significant in the workers exposed to pesticides *vs.* controls, (*p* ≤ 0.001) (nuclear buds (1.76 ± 0.19 vs. 0.57 ± 0.15), binucleated cells (8.75 ± 0.93 vs. 3.71 ± 0.41), cells with condensed chromatin (9.66 ± 1.00 vs. 2.25 ± 0.35), karyorrhexis (1.07 ± 0.15 vs. 0.038 ± 0.028), pyknosis (3.86 ± 0.63 vs. 1.24 ± 0.36), and karyolysis (2.45 ± 0.30 vs. 0.96 ± 0.22)). The results clearly demonstrated that exposure caused changes in cytokinesis, and lead to increased number of cells going to necrosis, but mostly in the early apoptosis. Associated sociodemographic variables explained variation of some parameters of these assays. Micronucleus frequencies was influenced by explicative variable “medication use” in the model, exposed participants who ingested medications were more likely to have this type of harm when exposed to pesticides (b = 0.993 ± 0.446, *p* = 0.031). In nuclear abnormalities in epithelial cells of the oral mucosa, variation in binucleated cells was influenced by age (b = 0.1741 ± 0.0897, *p* = 0.059), working period in years (b = −2.024 ± 0.84, *p* = 0.02 and medication (b = 4.16 ± 1.99, *p* = 0.042) in the model. Older participants, with the longest working years period and who reported taking medications had a greater probability of abnormality. In the same way, karyorrhexis was influenced on the explicative variable gender (male) (b = 1.434 ± 0.547, *p* = 0.012) and alcohol consumption (b = −0.1689 ± 0.0512, *p* = 0.002).

## 4. Discussion

Although there are cytogenetic biomonitoring studies performed in many different countries, and even though Mexico is a country with great agricultural activity with 8 million individuals dedicated to agricultural activity according to the results from 2015 [32], there are only few studies in this occupational setting in Mexico with controversial results. There are also no reports from Zamora-Jacona, Michoacan, although that region is one of the most important agricultural regions in Mexico, characteristic by the numerous species of different mites and botrytis, and therefore, by the simultaneous and increasing use of different pesticides. Since most of the pesticides used in this part of Mexico belong to organophosphate and carbamates that can influence the levels of cholinesterase in blood, the assessment levels of both type of cholinesterase (AChE and BChE) can give us an input on chronic or acute level exposure. With the fear of invasive blood technique as one of the reasons for the small number of workers in agricultural biomonitoring studies, there is a need to replace these methods with the less invasive ones, such as the buccal epithelial sampling.

Our objective was a determination of a classical biological indicator, the cholinesterases activities and additionally—cytogenetic damage, evaluated with comet assay, L-CBMNcyt test, and buccal MN cytome assay, in workers occupationally exposed to a mixture of pesticides and their controls living in the same region. In our study, we had 80 participants, among them 54 exposed workers. Although the exposure group was of the middle size, Bernieri et al. [20] already demonstrated that although small sample sizes can weaken a study, by making potential effects more difficult to detect, it does not undermine the validity of the observed significant differences [20]. This information was very crucial for us since we demonstrated that all DNA damage parameters were significantly elevated in the exposed group and that age, smoking, drinking period or even working period in years (from 1 to 10 years, mean value around 6 years) did not influence on the significance of the results.

Since workers were usually exposed to a complex mixture of pesticides, it was also crucial to determine the type of the pesticide and the group they belong to, to discover whether that fact will have an impact on our results and on the results of other biomarkers used in the study. Pesticide exposure was mostly represented by insecticides, herbicides, and fungicides, principally organophosphates (47%), carbamates (21%), organochlorines (16%) and pyrethoids (16%). First, two types to whom workers were mostly exposed, organophosphates and carbamates, can inhibit the level of cholinesterases in blood or in serum [33], and among the workers in our study the highest percentage of pesticides used by agricultural workers belong to these groups (70%). In our study, we did not find lower values of AChE and BChE in blood nor in serum of exposed workers. Our findings demonstrate that our workers were not recently exposed to the inhibitors of cholinesterase activity (according to the levels of BChE activity that has a period from the exposure after which it recovers from the inhibition) or that they were not exposed to these type of pesticide inhibitors in elevated levels (AChE and BChE activity). It could be also that occupational exposure to pesticides among farmers was not high enough to inhibit cholinesterases, or that non-farmers might be exposed to pesticides from the environment (our controls were living in the same area as the farmers and could be indirectly exposed to pesticides). Since the use of PPE can influence on the negative association between AChE and BChE activities depletion and pesticide exposure [34], the use of protective measurements among most of the workers as in the study of Taghavian et al. [35] has certainly influenced the results. Although we could not compare the acetylcholinesterase levels with other studies due to different measurement units and types, we have found many articles that confirmed our results such as the work of Krieger and Dinoff [36], or articles that demonstrated even unchanged or nonsignificant changes in the exposed and control group [37,38], even though various studies have demonstrated the inhibitory effect of organophosphate and carbamate pesticides on cholinesterase activities. At the end, previous results from other colleagues in our group demonstrated that determination of AChE activity does not need to provide reliable evidence for exposure and that the use of other test or biomarkers to detect effects of long-term exposure to pesticides should be recommended [15]. It seems that determination of cholinesterase inhibition is useful in acute poisoning case monitoring or high-levels exposure [15], but is not predictive in chronic or delayed effects such as the assessment of DNA damage, that can be also a consequence of the elevated levels of reactive oxygen species or reactive nitrogen species connected with the pesticide exposure [39,40]. As a confirmation of these conclusions are the results of other authors who demonstrated elevated levels of DNA damage in unchanged AChE and BChE levels after exposure (for example in sisters’ chromatid exchange assay [41], or no correlation between exposure time and DNA damage and cholinesterase activity [20]).

The most used methods for DNA damage biomonitoring in human peripheral blood to study effects of environmental or occupational exposure to (putative) carcinogens are comet and micronucleus assay [42], and they have been also used to determine the extent of DNA damage in workers with occupational exposure to pesticides. Although very difficult to attribute the effects found to any product, it has been demonstrated that occupational exposure to the pesticide mixtures induce increase in DNA damage levels in both type of the assay [17,18,43,44,45,46,47,48,49,50]. Most of the pesticides to which farmers are exposed, such as organophosphates, organochlorines, carbamates and pyrethroids, have been found capable of generating oxidative stress, as one of the main causes of DNA damage [49].

In our study, all four parameters of primary DNA damage in comet assay had significantly elevated levels of DNA damage compared to the control group, with the following ratios: tail moment (3.35 times higher), Olive tail moment (2.86 times higher), tail intensity (2.43 times higher) and tail length (1.42 times higher than control values).

The results implicate that although acetylcholinesterase measurements did not demonstrate satisfying results (no inhibition in the exposed group), the comet assay method was sensitive enough to detect DNA damage, even when the pesticides were used in their regular concentrations and the exposure of the workers was not extremely high. Nonetheless, this also demonstrated how important the pesticide risk assessment is even when there is a low dose exposure, no matter of the working period in years (in our case from 1 to 10-year, mean value 6 years), and that pesticides can cause genomic instability, first seen on the primary, repairable level.

Furthermore, it is important that we demonstrated that MN frequency in lymphocytes (representing systemic DNA damage due to the lymphocyte circulation through the body) and in buccal epithelial cells (as the first level of exposure after inhalation or ingestion of pesticide) has the same ratio (2.88) between spontaneous micronucleus frequency level found in control group and the exposed group, since it is the manifest that one invasive blood method could be complemented or even replaced with certainty with one noninvasive method in buccal samples. Although the numbers from two assays that are measuring permanent DNA damage cannot be compared (but the ratios can), this is an interesting observation that confirms our earlier findings on the correlation curve between MN found in lymphocytes and in the buccal cells [51,52]. Although according to our previous results on the spontaneous frequency of buccal MN (from 0.32 to 1.70 on 1000 differentiated cells), one could argue that exposed workers still have normal values for MN buccal frequency, significantly higher values from the control in both assays gives us the right to say that these results do have a real value. It is also interesting to mention that repair mechanisms are not active in buccal cells, meaning that the initial damage will not be lost in one part during the repair process (the process that can happen in L-CBMNcyt), and the fact that the ratio is the same, means that type of DNA damage in lymphocyte is also permanent and not lost during repair. These facts only prove that in the risk assessment, L-CBMNcyt could be complemented or even replaced with noninvasive method, which would certainly attract more volunteers who are usually afraid of invasive techniques. In other studies, the ratio between MN lymphocytes in exposed and control group ranged from 1.12–15.8 [53]. Since our control group was from the same region, our 2.88 ratio in both assays, in range as in other studies, demonstrates that there is a genotoxic risk present among agricultural workers even when they are using the protective equipment (87% in our study). Significantly higher level of genomic instability in agricultural workers was seen in the frequency of nuclear buds or so-called broken eggs and in the frequency of binucleated cells. Significantly elevated levels of nuclear buds and broken eggs are considered as a biomarker for amplified DNA and/or DNA repair complexes elimination and can play an important role in tumor progression [49,54].

Significantly elevated levels of binucleated cells, as a biomarker cell proliferation of alterations, demonstrated that genomic instability [49] is already permanent (implemented) on the basal cells level, where cells are becoming epithelial, after their last division before becoming differentiated. It has been suggested that binucleated cells are the first step before the cell becomes higher-ploidic, rather than aneuploid as suggested before, and ploidy could play a role in the malignant transformation of a given cell population [55]. It seems that in cytokinesis checkpoint for aneuploidic binucleated cells, non-disjunction occurs with a higher frequency in binucleated cells that fail to complete cytokinesis rather than in cells that have completed cytokinesis, yielding rather tetraploid and not aneuplodic cells [56]. Since most of the tumors and cancers are of epithelial origin and since buccal mucosa is the site for oral cancer, this demonstration of elevated levels of genomic instability is a cause for concern for genotoxic and cancer risk assessment [57]. This fact has been also confirmed among people with Down syndrome, who also (as in our study) demonstrated changes in cells with condensed chromatin, karyorrhectic cells, karyolitic and pyknotic cells [58].

Buccal micronucleus parameters demonstrated significantly elevated levels of cells in necrotic and apoptotic phase, and most of them in the exposed group were in the phase of early apoptosis, confirmed with the frequency of the cells with condensed chromatin. Apoptosis, or programmed cell death, is usually activated when the genomic instability is too high for the cell to bear the damage and when the organism wants to eliminate this type of cells rapidly.

We have compared our results with existing studies that used the same biological indicators in humans to assess the exposure and the risk to pesticides, but their number is very small, although there is a great potential in combining and analyzing these specific biomarkers. Different design of the studies, including small number of participants in the studies, makes it even difficult to compare the results from those studies. Beside the small participants number, there are differences in the geographic and meteorological characteristics of the agricultural areas and countries directly affecting the agricultural workers exposure and influencing the results due to exposure level differences, differences in the used pesticides types and differences in the used pesticides frequencies and even in the duration of the exposure, referring to the hours per day and years of working with the pesticides and also to the use of protection equipment [59].

Garaj-Vrhovac and Želježić [60], showed a good agreement between micronucleus, chromosomal aberrations, sister chromatid exchanges and comet assays, in a cytogenetic monitoring of a population occupationally exposed to a complex mixture of pesticides, like Da Silva et al. [18] and Jonnalagadda et al. [61], with micronucleus and comet assay. On the other hand, disagreement results were found by Želježić et al. [15], Pasquini et al. [62], and Joksić et al. [63], who showed increases in the micronucleus frequency and negative results with sister chromatid exchanges or DNA damage detected with comet assay.

The above reflects that different parameters are being evaluated, product of chronic or recent exposures [12] points to the important differences between the three tests, and supports the idea that these assays should be used simultaneously as complementary methods for genotoxicity assessment [64].

Although not shown in the results, an interesting observation was the connection between BMI index and DNA damage found in this study. According to the multiple linear regression models (in which it is detected that independent variables have a great effect on the variation of a dependent one) it was found that for the tail moment in the comet assay the only independent variable that contributes to its variability is the BMI (b = 0.3161 ± 0.156, *p* = 0.049). Arévalo-Jaramillo et al. [38], in their study over biochemical and genotoxic effects in women exposed to pesticides in Southern Ecuador, observed that the population with highest rate of obesity also exhibited greater genotoxic effects. Our findings could be a consequence of the ability of some pesticides to accumulate in fatty tissue [65], thus overweight or obesity may be a factor that can increase the risk of intoxication from pesticides and the level of oxidative stress that causative can also influence on the level of DNA damage [66].

Connections between obesity, body mass index, waist circumference and higher DNA damage have been reported in many studies, and some of them in vivo and on humans that are connected with the comet assay, micronucleus (buccal and lymphocytes assay) and other DNA damaging assessment assays can be found in the review of Setayesh et al. [67]. There are reports that broad range of DNA lesions such as double strand breaks (DSB), single strand breaks (SSB) or oxidized bases can be even about two-times higher. BMI linked always with obesity is connected with chronic energy overload resulting in enhanced ROS production and inflammation. Chronic inflammation is strongly associated with the formation of DNA lesions, which can be usually changed with the decrease after caloric reduction diet. Increased body weight is connected with disturbances in DNA damage response pathway, there is an inverse association between BMI and nucleotide excision repair (NER) capacity, and obesity and therefore BMI can alter the repair of DSBs induced by genotoxic agents. Obesity-associated enhanced ROS production can modulate the DNA damage response, change expression of genes involved in DNA repair, inhibit DNA repair enzymes, and alter gene expression related to response to stress and toxic agents.

Although there are studies reporting that with the use of protective equipment, there is no increase in DNA damage from the pesticides exposure [68,69,70], we would hardly believe that 87% of participants in our study have lied to wear the protective equipment, and we do not accept that misuse or not wearing PPM and lying about that would explain significantly elevated levels of DNA damage in all three test systems applied, and in the same time not to have also inhibition in acetylcholinesterase levels. Carbajal-López et al. [44] as in our study also demonstrated that exposure did not demonstrate correlation with DNA damage found in comet assay nor in micronucleus assay in buccal cells in Mexican agricultural workers from Guerrero. In our study, neither smoking, drinking habits, gender, age, nor antiquity did no affect the results for DNA damage when compared in single linear model, but included in multiple linear models, there was an effect of BMI, age, antiquity, medication, sex and alcohol consumption, with the results suggesting that karyorrhexis model was the more explicative (R2 value 28%), and the low values of R2 for other models indicates that the influence of independent variables was low and must be carefully considered. In addition, the study of Carbajal-López et al. [44] demonstrated the same, signifying that confounding factors should always be consider when performing a genotoxic evaluation, but with cautions with the results interpretation, since the potential influence of the confounding factors (smoking, drinking or dietary habits, including even the weight) on the DNA damage caused by pesticides is still under investigation and is quite difficult to be accurately determined [43,71]. The biomonitoring of pesticide effects in agricultural workers is therefore complicated, but critical for their protection, elucidating the risk associated with exposure to specific compounds or complex mixtures, as well as pesticide-related occupations or specific cultivation practices but also in assessment of acute or chronic exposure levels [12].

We would expect that age would correlate with the level of DNA damage as in our other studies [72], but the lack of correlation is certainly connected with the small group of participants and the fact that mean value of age was less than 40 years. Therefore, the use of noninvasive methods such as buccal sampling, on which also micronucleus assay and comet assay could be performed [44] would for sure attract more individuals for sampling, and therefore statistical power of the future studies would be much stronger with more age group representatives.

## 5. Conclusions

The combined use of biochemical and cytogenetic techniques can be useful to assess the risk of pesticide exposure.

Human chronic exposure to pesticide mixtures may result in increased DNA damage in the absence of alteration in the levels of acetylcholinesterase activity, suggesting a steeper dose response relationship for genotoxicity. Buccal sampling has been again confirmed as the valuable noninvasive method and replacement biomarker for the blood sampling genotoxic techniques.

The outcome of the study revealed a relation between pesticide exposure and increase in the level of DNA damage measured by comet assay and MN test. The last has been associated to the risk of cancer. Thus, a higher level of genetic damage may imply consequently higher risk of cancer and other non-communicable diseases.

Initiation of awareness campaigns and training to educate them with the use of PPE, effective personal hygiene and post exposure cleanliness would minimize the deleterious effects of pesticide exposure.

## Figures and Tables

**Table 1:** Characteristics of the control and the exposed group.

Characteristics	Control Group		Exposed Group	
*n*	26		54	
Gender (M/F) (%)	8/18	(31/69)	52/2	(96/4)
Age (years, mean ± SD) (range)	37.62 ± 3.10	(19–72)	36.09 ± 1.60	(18–71)
BMI (kg/m^2^, mean ± SD) (range)	26.05 ± 1.06	(18.51–39.89)	28.49 ± 0.65	(17.90–41.91)
Exposure time (in years, mean ± SD) (range)	NA		5.36 ± 0.43	(1 ≤ 10)
*Smoking*				
Smokers, n (%)	3	(12)	17	(31)
Non-smokers, n (%)	23	(88)	37	(69)
*Alcohol intake*				
yes, n (%)	2	(8)	30	(56)
no, n (%)	24	(92)	24	(44)
PPM, n (%)	NA		47 of 54	(87)

*n*—number of volunteers, M—male, F–female, SD—standard deviation, BMI—body mass index, PPM—personal protective measures (equipment and procedure), NA—not applicable.

**Table 2:** List of the more frequent pesticides used by the exposed group and their hazard classification.

P	CC	Compound	IUPAC Name	WHO	USEPA/IARC
I	Organophosphate	Diazinon	O,O-diethyl O-[4-methyl-6-(propan-2-yl)pyrimidin-2-yl] phosphorothioate	II	NLC/Group 2A
		Dimethoate	O,O-dimethyl S-[2-(methylamino)-2-oxoethyl] dithiophosphate	II	Group C/NE
		Gusathion (Azinphos-ethyl)	O,O-diethyl S-[(4-oxo-1,2,3-benzotriazin-3(4H)-yl)methyl] phosphorodithioate	Ib	NE
		Lorsban (Chlorpyrifos)	O,O-diethyl O-3,5,6-trichloropyridin-2-yl phosphorothioate	II	Group E/NE
		Malathion	Diethyl 2-[(dimethoxyphosphorothioyl)sulfanyl]butanedioate	III	SEC/Group 2A
		Orthene (Acephate)	N-(methoxy-methylsulfanylphosphoryl)acetamide	II	Group C/NE
		Parathion (Folidol)	O,O-diethyl O-(4-nitrophenyl) phosphorothioate	Ia	Group C/Group 2B
		Tamaron (Methamidophos)	O,S-dimethyl phosphoramidothioate	Ib	NLC/NE
	Carbamate	Furadan (Carbofuran)	2,2-dimethyl-2,3-dihydro-1-benzofuran-7-yl methylcarbamate	Ib	NLC/NE
		Lannate (Methomyl)	(E,Z)-methyl N-{[(methylamino)carbonyl]oxy}ethanimidothioate	Ib	Group E/NE
		Vydate (Oxamyl)	Methyl 2-(dimethylamino)-N-[(methylcarbamoyl)oxy]-2-oxoethanimidothioate	Ib	Group E/NE
	Organochlorine	Aldrin	1,2,3,4,10,10-hexachloro-1,4,4a,5,8,8a-hexahydro-1,4:5,8-dimethanonaphthalene	O	Group B2/Group 3
		Dicofol (Kelthane)	2,2,2-trichloro-1,1-bis(4-chlorophenyl)ethanol	II	Group C/Group 3
		Endosulfan	6,7,8,9,10,10-hexachloro-1,5,5a,6,9,9a-hexahydro- 6,9-methano-2,4,3-benzodioxathiepine-3-oxide	II	NLC/NE
	Piretroides	Baytroid (Cyfluthrin)	β-cyfluthrin Cyano{4-fluoro-3-phenoxyphenyl)methyl-3-{2,2-dichloroethenyl)-2,2-dimethyl-cyclopropanecarboxylate	1b	NLC/NE
		Karate (Lambda-cyhalothrin)	(R)-α-cyano-3-phenoxybenzyl (1S)-cis-3-[(Z)-2-chloro-3,3,3-trifluoropropenyl]-2,2-dimethylcyclopropanecarboxylate and (S)-a-cyano-3-phenoxybenzyl (1R)-cis-3-[(Z)-2-chloro-3,3,3-trifluoropropenyl]-2,2-dimethylcyclopropanecarboxylate	II	Group D/NE
		Talstar (Bifenthrin)	2-Methyl-3-phenylphenyl)methyl (1S,3S)-3-[(Z)-2-chloro-3,3,3-trifluoroprop-1-enyl]- 2,2-dimethylcyclopropane-1-carboxylate	II	Group C/NE
H	Organophosphate	Paraquat	1,1′-dimethyl-4,4′-bipyridinium dichloride	II	Group C/NE
F	Carbamate	Manzate (Mancozeb)	Zinc;manganese(2+);N-[2-(sulfidocarbothioylamino)ethyl]carbamodithioate	U	Group B/Group 2B

I—insecticides, H—herbicides, F—fungicides, P—pesticides, CC—chemical class, IUPAC—International Union of Pure and Applied Chemistry, NE—not evaluated, WHO—classification: Ia—extremely hazardous; Ib—highly hazardous; II—moderately hazardous; III—slightly hazardous; U—unlikely to present acute hazard in normal use; FM—fumigant, not classified; O—obsolete as pesticide, not classified [28]. USEPA Cancer Classification: NLC—not likely to be carcinogenic to humans; SEC—suggestive evidence of carcinogenicity; Group B2—probable human carcinogen; Group C—possible human carcinogen; Group D—not classifiable as to human carcinogenicity; Group E—evidence of non-carcinogenicity for humans [29]. IARC: Group 1—carcinogenic to humans; Group 2A—probably carcinogenic to humans; Group 2B—possibly carcinogenic to humans; Group 3—not classifiable as to its carcinogenicity to humans [30].

**Table 3:** Comparison of cholinesterases activities in study population.

	AChE Whole Blood		AChE Serum		BChE Serum	
Parameter	Subjects		Subjects		Subjects	
	Unexposed	Exposed	Unexposed	Exposed	Unexposed	Exposed
Mean (U/mL)	4.02	4.73	35.32	52.35	231.76	296.73
Maximum	6.68	7.11	20.18	79.83	380.35	414.71
Minimum	1.40	2.38	55.07	34.98	118.86	204.26
SD	1.40	1.23	11.07	10.04	81.60	60.78
		*p* > 0.16		*p* < 0.0006		*p* < 0.0047
AChE—Acethylcholinesterase, BChE—Buthyrilcholinesterase						

**Table 4:** Human biomonitoring of agricultural workers from Zamora-Jacona in Michoacan Mexico with different cytogenetic assays.

Assay/Parameters	Control Group (*n* = 26)	Exposed Group (*n* = 54)	Level of Significance (*p*)
**Comet Assay**			
Tail Length,µm	55.62 ± 13.88	78.80 ± 25.00	<0.0001
Tail Intensity, %	9.21 ± 4.10	22.40 ± 9.82	<0.0001
Tail Moment	1.89 ± 1.24	6.34 ± 5.02	<0.0001
Olive Tail Moment	0.24 ± 1.18	6.31 ± 9.73	<0.0001
**Cytokinesis-block micronucleus cytome assay**			
MN	2.68 ± 4.35	7.53 ± 5.45	<0.001
**Buccal micronucleous cytome assay**			
MN	0.269 ± 0.365	0.777 ± 1.39	<0.05
Binucleated cells	3.712 ± 2.069	8.754 ± 6.43	<0.001
Karyorrhectic cells	0.038 ± 0.141	1.067 ± 1.07	<0.001
Pyknotic cells	1.243 ± 1.841	3.863 ± 4.34	0.001
Karyolitic cells	0.962 ± 1.110	2.449 ± 2.09	<0.001
Nuclear Buds	0.571 ± 0.744	1.762 ± 1.30	<0.001
Condensed Chromatin	2.252 ± 1.785	9.662 ± 6.91	<0.001

Values represent Mean ± SD of frequencies per 1000 cells or calculated frequencies on 1000 binucleated cells (for MN and nuclear buds) from 2000 binucleated cells counted for those parameters, MN-micronucleus, Statistical analysis was conducted with Mann–Whitney U-test.
